# Differences between Physicians

**Published:** 1850-05

**Authors:** 


*Of differences between Physicians.*—Diversity of opinion, and opposition of interest, may, in the medical, as in other professions, sometimes occasion controversy and even contention. Whenever such cases unfortunately occur, and cannot immediately be terminated, they should be referred to the arbitration of a sufficient number of physicians, or a *court-medical.*

As peculiar reserve must be maintained by physicians towards the public, in regard to professional matters, and as there exist numerous points in medical ethics and etiquette through which the feelings of medical men may be painfully assailed in their intercourse with each other, and which cannot be understood or appreciated by general society, neither the subject matter of such differences nor the adjudication of arbitrators should be made public, as publicity in a case of this nature may be personally injurious to the individuals concerned, and can hardly fail to bring discredit on the faculty.

To return to the subject of *demonstrative midwifery*, so far as expressions of opinion have come to our knowledge, they go to sustain the belief hazarded by us in the February number of this Journal, that the "plan must commend itself to the approbation of the medical profession." But whether we were correct in this assertion or not, does not necessarily affect the merits of the plan itself. The latter we design to consider, at length, at some future date, provided we are not anticipated by some one more capable than ourselves of doing justice to the subject.

